# Patient-specific beta-tricalcium phosphate scaffold for customized alveolar ridge augmentation: a case report: Case Report: patient-specific β-TCP scaffold for alveolar ridge CBR

**DOI:** 10.1186/s40729-024-00541-2

**Published:** 2024-05-01

**Authors:** Daphne Schönegg, Harald Essig, Adib Al-Haj Husain, Franz E. Weber, Silvio Valdec

**Affiliations:** 1 Department of Oral and Maxillofacial Surgery, University Hospital Basel, Spitalstrasse 21, Basel, 4031 Switzerland; 2 Department of Oral and Maxillofacial Surgery, University Hospital Zurich, Frauenklinikstrasse 24, Zurich, 8091 Switzerland; 3 Clinic of Cranio-Maxillofacial and Oral Surgery, Center of Dental Medicine, University of Zurich, Plattenstrasse 11, Zurich, 8032 Switzerland

**Keywords:** beta-tricalcium phosphate, β-TCP, Guided bone regeneration, Customized bone regeneration, CBR, Two-stage implant therapy, Case report

## Abstract

**Background:**

Beta-tricalcium phosphate (β-TCP) is a biocompatible ceramic material widely used in the field of oral regeneration. Due to its excellent biological and mechanical properties, it is increasingly utilized for alveolar ridge augmentation or guided bone regeneration (GBR). With recent advances in computer-aided design and manufacturing (CAD/CAM), β-TCP can now be used in the form of digitally designed patient-specific scaffolds for customized bone regeneration (CBR) of advanced defects in a two-stage implant therapy concept. In this case report following the CARE case report guidelines, we present a novel application of a patient-specific β-TCP scaffold in pre-implant mandibular alveolar ridge augmentation.

**Case presentation:**

A 63-year-old female patient with significant horizontal bone loss in the posterior mandible was treated with a custom β-TCP scaffold in the context of a two-stage backward-planned implant therapy. Cone-beam computed tomography nine months after augmentation showed successful integration of the scaffold into the surrounding bone, allowing implant placement. Follow-up until two years after initial surgery showed excellent oral and peri-implant health.

**Conclusions:**

This case highlights the potential of patient-specific β-TCP scaffolds for alveolar ridge augmentation and their advantage over traditional techniques, including avoidance of xeno-, allo-, and autografts. The results provide encouraging evidence for their use in clinical practice. Patient-specific β-TCP scaffolds may be a promising alternative for clinicians seeking to provide their patients with safe, predictable, and effective alveolar ridge augmentation results in customized bone regeneration procedures.

**Supplementary Information:**

The online version contains supplementary material available at 10.1186/s40729-024-00541-2.

## Background

Alveolar ridge augmentation is often needed prior to implant placement when bone defects prevent a functionally or esthetically ideal dental implant placement. Various materials and techniques have been described for alveolar ridge augmentation of advanced defects in two-stage implant therapy concepts [1, 2]. Guided bone regeneration (GBR) with alloplastic bone graft substitutes has become a standard approach to treating such bone defects, avoiding the drawbacks of xenografts, allografts, and autografts. Beta-tricalcium phosphate (β-TCP) offers excellent biological and mechanical properties, including favorable resorption characteristics, osteoconductivity, and stability. To date, it has mainly been used in the form of granules or blocks. With recent advances in computer-aided design and manufacturing (CAD/CAM), β-TCP can now also be utilized to fabricate patient-specific scaffolds via additive manufacturing, enabling its use in customized bone regeneration (CBR). CBR allows oral surgeons to provide precise, predictable, and effective alveolar ridge augmentation solutions for each patient with reduced intra-operative technical demands, even in the most complex cases where conventional techniques may not be the first choice.

This case report presents a unique application of a patient-specific β-TCP scaffold for customized mandibular alveolar ridge augmentation using preoperative cone-beam computed tomography for prosthetically driven digital scaffold design. It aims to demonstrate the potential of such patient-specific scaffolds as a safe and successful approach to CBR.

## Case presentation

A 63-year-old woman with an unremarkable medical history presented to our department to have her missing tooth 46 replaced. The right mandibular first molar had been extracted approximately one year previously due to extensive caries. Clinical examination revealed good oral hygiene and no evidence of periodontal disease. The neighboring teeth were intact and healthy, and the single-tooth gap was of sufficient size, so a dental implant was the preferred option for tooth replacement. Radiographic examination using cone-beam computed tomography (CBCT) confirmed an extensive horizontal bone defect in the right posterior mandible, indicating the need for pre-implant alveolar ridge augmentation to restore both the height and width of the ridge. It was determined that patient-specific ridge augmentation with a resorbable scaffold would be a valid option for predictable three-dimensional bone regeneration in this case.

**Fig. 1:**
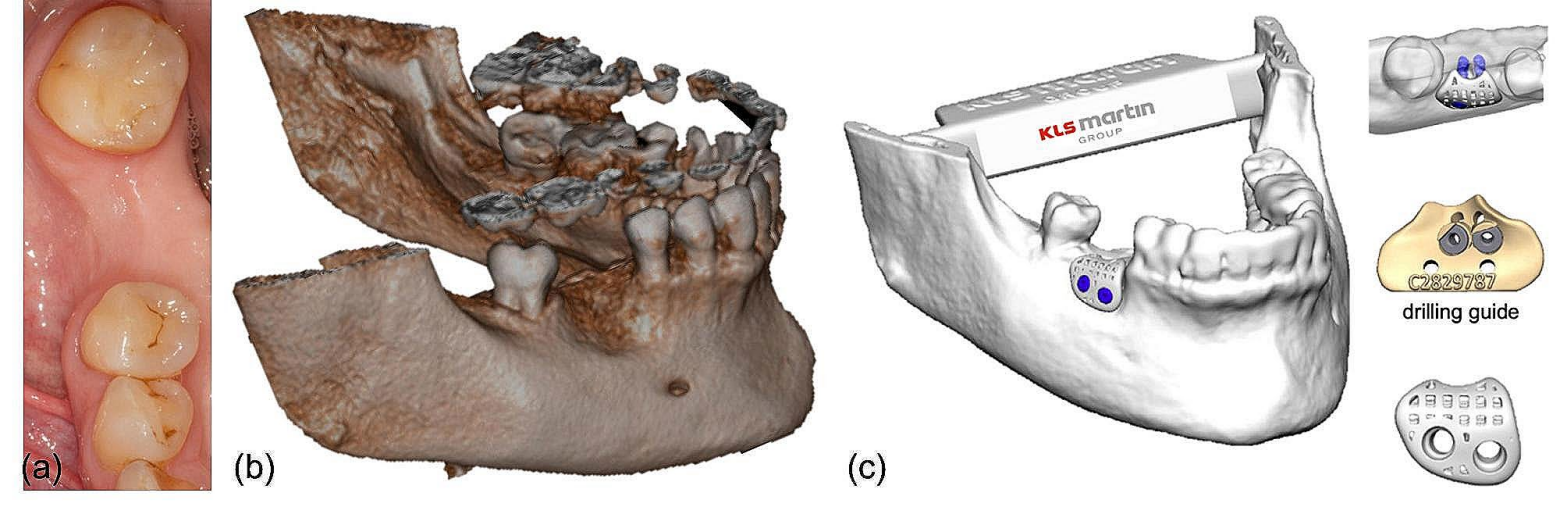
Pre-operative clinical (left) and radiologic (middle) situation demonstrating extensive horizontal and vertical bone defect after extraction of the right mandibular first molar. Right: digitally planned beta-tricalcium phosphate (β-TCP) scaffold with the matching drilling guide

Based on the initial CBCT scan (3D Accuitomo 370, Morita, Dietzenbach, Germany) and an intraoral scan (3Shape TRIOS, Straumann, Basel, Switzerland), prosthetically driven implant planning was performed in SMOP (Swissmeda, Baar, Switzerland). A β-TCP scaffold was digitally designed by specialized engineers (KLS Martin Group, Tuttlingen, Germany) following the manufacturer’s certified and patented workflow to reconstruct the contour of the alveolar ridge and to meet both anatomical/esthetic and functional requirements. Holes for the fixation screws and a recess at the future implant position were left free in the scaffold. The design of the scaffold and the position of the screw holes were evaluated and approved by the oral surgeon in the design and communication software (IPS Gate®, KLS Martin). Matching drill guides were fabricated to facilitate surgery and ensure the correct positioning of both the scaffold and the dental implant. Figure 1 shows the pre-operative clinical and radiologic findings and the digital planning.

**Fig. 2:**
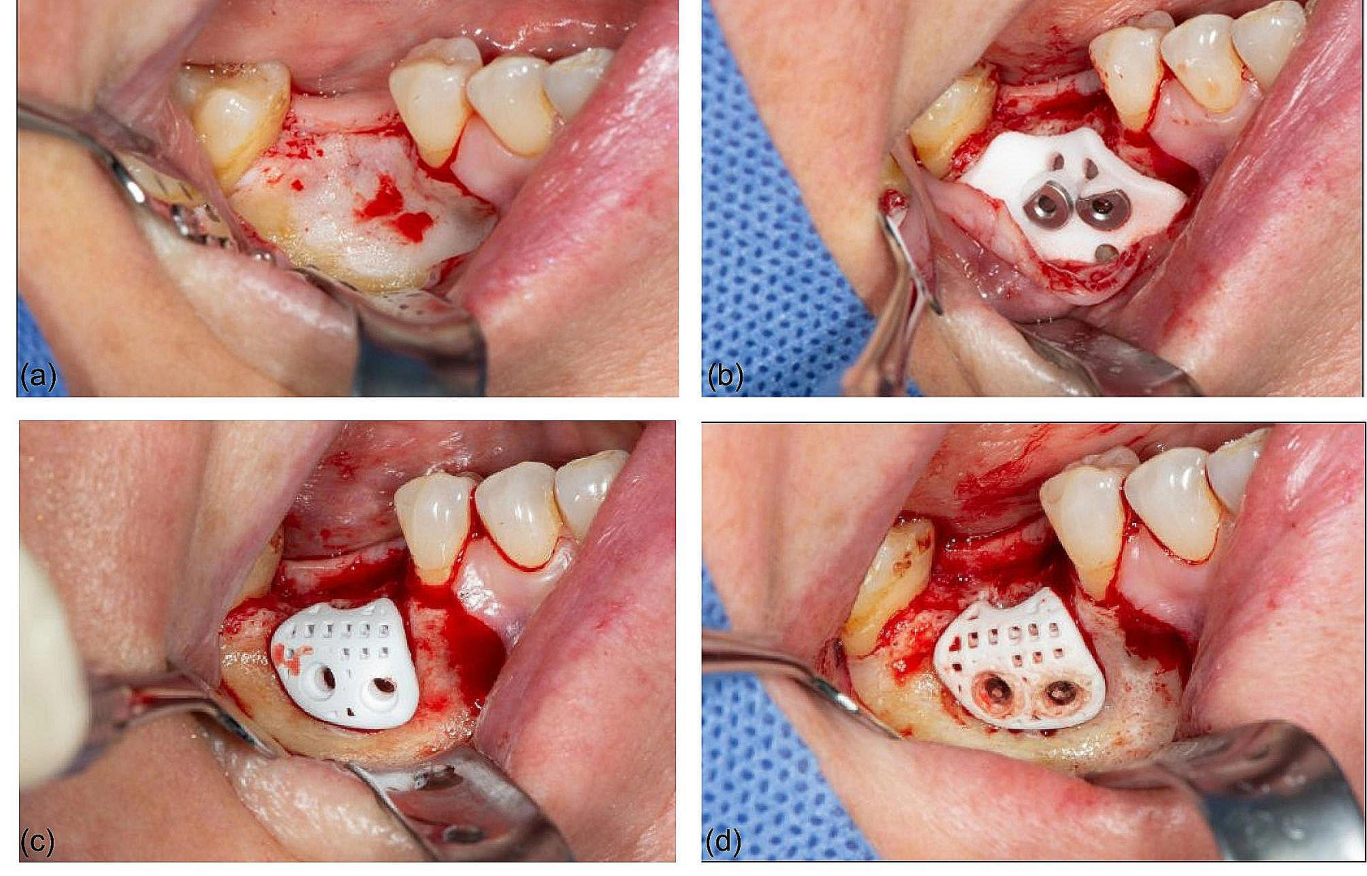
Intraoperative photographs demonstrating the bony defect (**a**), placement of the drilling guide (**b**), positioning of the custom beta-tricalcium phosphate (β-TCP) scaffold (**c**) and its fixation with resorbable poly-lactide pins (**d**)

The surgery was performed under local anesthesia as documented in Fig. 2. A mucoperiosteal flap was elevated in the right posterior mandible, exposing the bony defect. The drill guide was placed on the alveolar crest and two fixation holes were pre-drilled in the mandible. The custom β-TCP scaffold was then carefully positioned over the bony defect. The patient-specific design allowed unmistakable positioning of the scaffold according to the preoperative planning. The scaffold was secured with two resorbable poly-lactide pins (SonicWeld Rx®, KLS Martin Group). The gap between the scaffold and the mandible was loosely filled with autologous bone harvested locally with a bone scraper (Safescraper, Meta Biomed, Mülheim an der Ruhr, Germany) and the scaffold was covered with a collagen membrane (Jason® membrane, Straumann) to support the barrier function. The surgical site was closed with interrupted non-resorbable sutures after irrigation and hemostasis. The patient was prescribed non-steroidal anti-inflammatory drugs and antibiotics for five days postoperatively and was encouraged to maintain excellent oral hygiene. Stitches were removed after ten days when complete wound healing was assured.

**Fig. 3:**
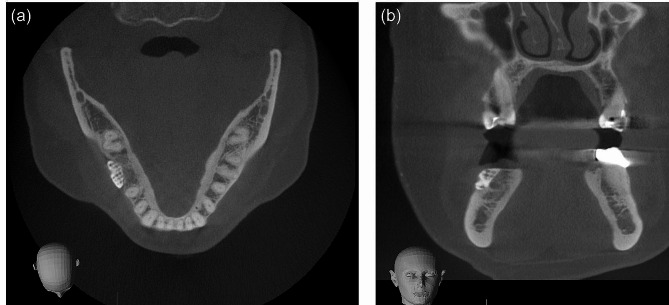
Post-operative axial (**a**) and coronal (**b**) reconstruction of the patient’s cone-beam computed tomography (CBCT) scan showing successful customized bone regeneration (CBR). Note that the structure of the beta-tricalcium phosphate (β-TCP) scaffold is still visible

Follow-up visits were held at regular intervals and showed uneventful healing. CBCT scans were performed to evaluate the healing process and to assess the alveolar ridge augmentation outcome. The CBCT scan nine months after surgery showed successful augmentation with a well-integrated β-TCP scaffold and adjacent new bone formation, now allowing for dental implant placement (Fig. 3).

**Fig. 4:**
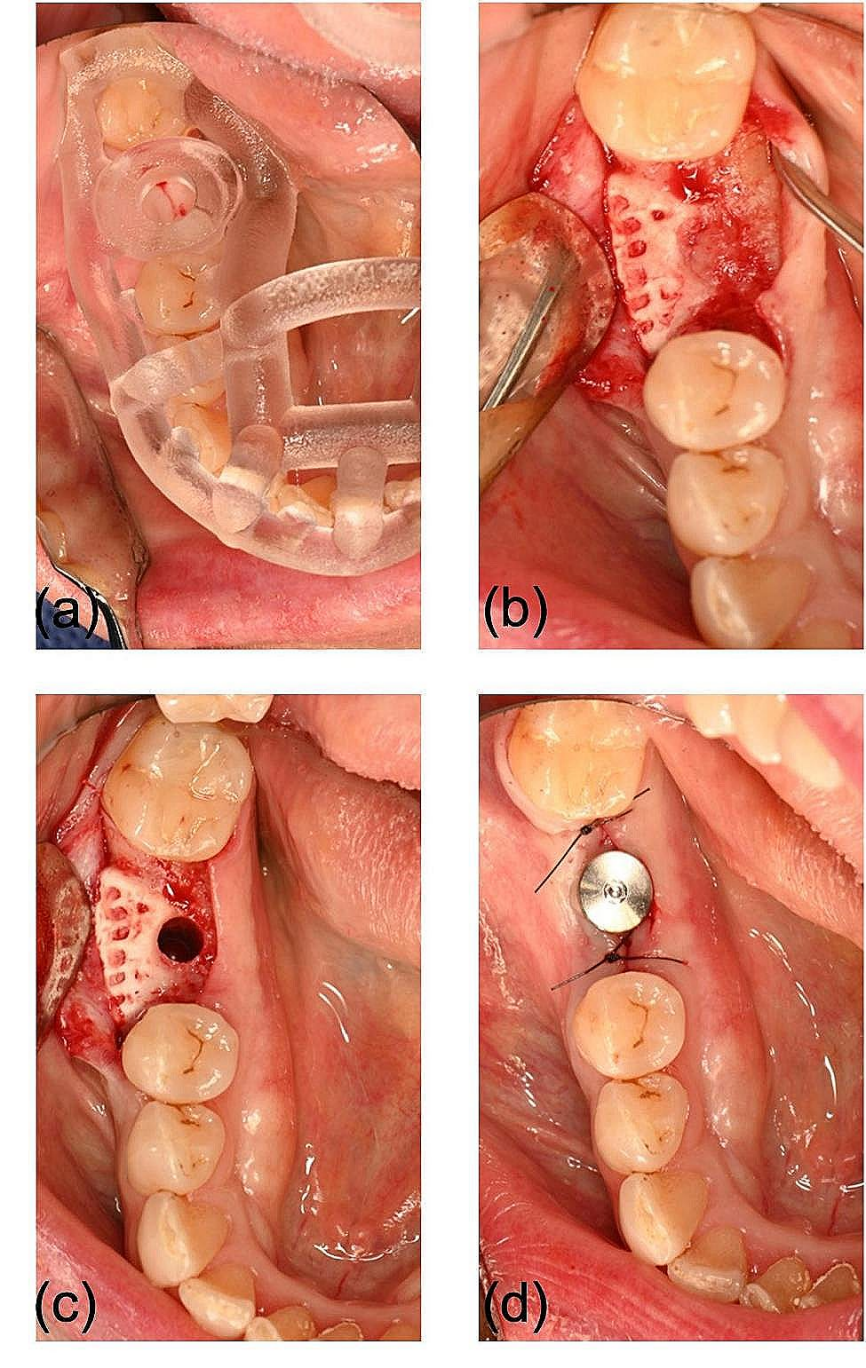
Intraoperative photograph showing the 3D-printed implant guide (**a**). Re-entry proved sufficient bone regeneration (**b**). After pre-drilling (**c**), the implant was placed with good primary stability and left for transmucosal healing (**d**)

**Fig. 5:**
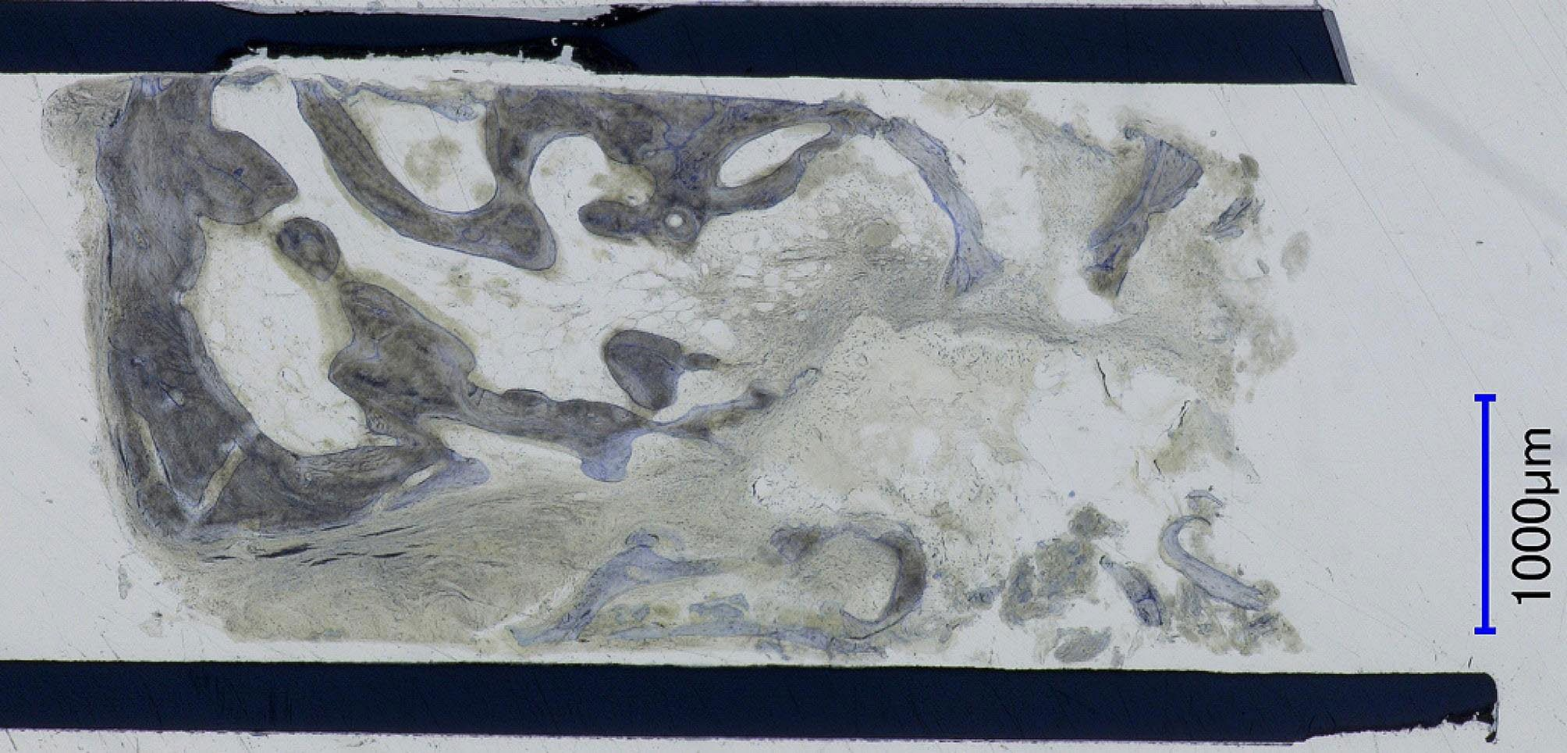
Histological examination of the drilled-out bone cylinder. Newly formed trabecular bone is stained blue, newly formed osteoid appears light gray, while preexisting bone prior to augmentation is dark gray

In a second surgical procedure under local anesthesia, the dental implant (Straumann SP, Ø 4.1 mm RN, 10 mm length) was placed in the previously digitally defined position with a 3D-printed implant guide with good primary stability after re-exposure of the crest. The implant was covered with a healing abutment and left for transmucosal healing (Fig. 4). The bone cylinder drilled out during implant placement was histologically examined and showed high-quality cancellous bone in the previously atrophied ridge, demonstrating the efficacy of the β-TCP scaffold for bone regeneration (Fig. 5).

**Fig. 6:**
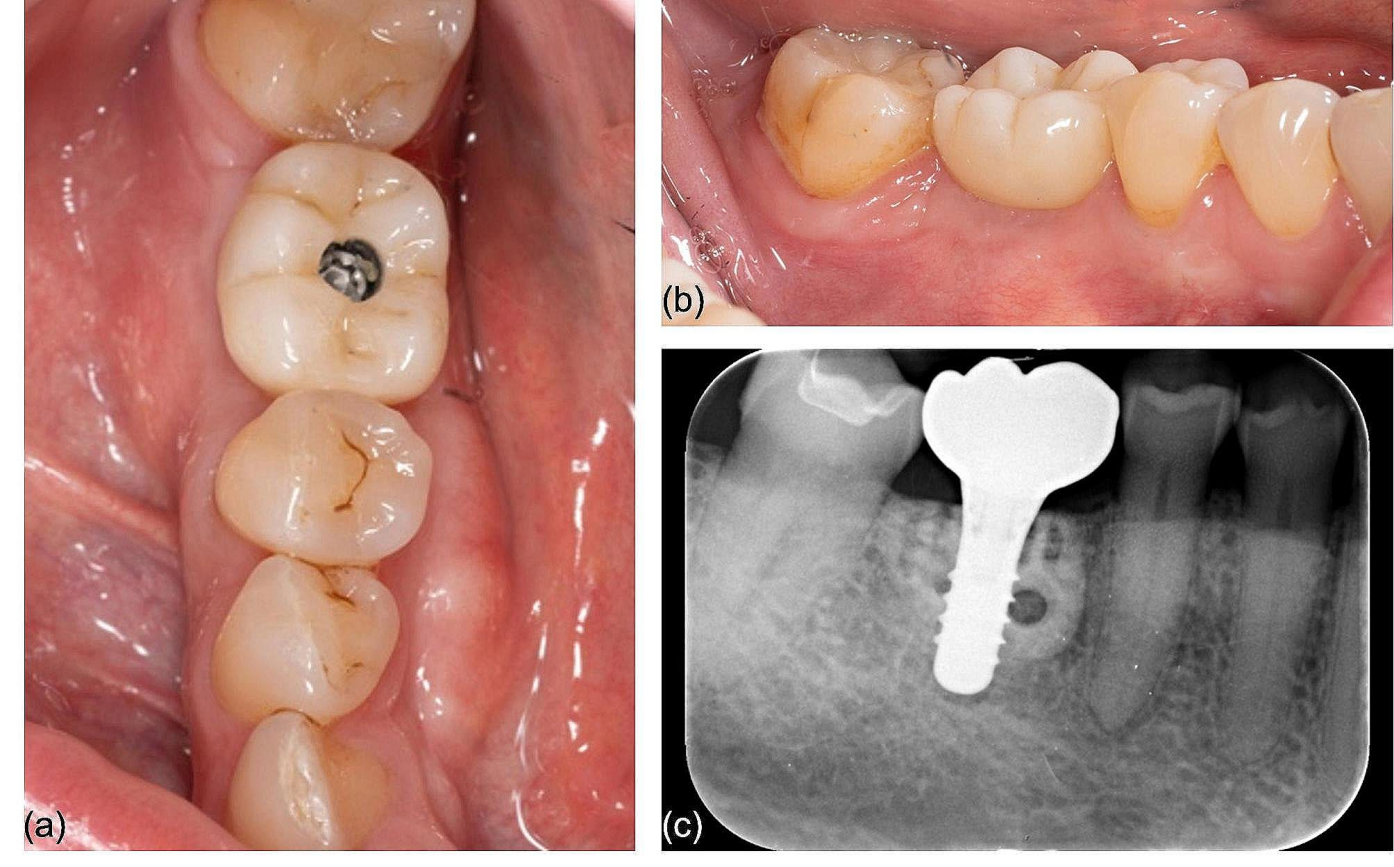
Post-operative clinical (**a**, **b**) and radiologic (**c**) situation

After complete bone integration 6 months post implant placement, the customized crown (milled zirconia framework veneered with aesthetic ceramic) was placed on the implant with a stock titanium abutment. Follow-up clinical examinations and radiographs showed good implant position with no evidence of peri-implant bone loss. The overlay of the recent CBCT with the digital planning showed a complete overlap of the contours, underlining the predictability of the technique. The patient was completely satisfied with the result and two years after the initial surgery now has excellent oral and peri-implant health (Fig. 6).

## Conclusions

As documented in case series and feasibility studies, customized bone regeneration (CBR) offers several advantages over traditional ridge augmentation techniques [3, 4]. Key advantages of CBR include improved accuracy and predictability of the augmentation result as the stable scaffold facilitates three-dimensional augmentation and helps to maintain the required space, improving the esthetics and function of the final restoration [5, 6]. The unique patient-specific fit allows for unmistakable scaffold placement during surgery, facilitating and shortening the procedure, and potentially reducing complication rates [7–9].

CBR procedures to date have mostly been performed with non-resorbable CAD/CAM titanium meshes requiring a second surgery to remove the hardware before implant placement, or with allografts associated with drawbacks such as variable quality, graft resorption, and ethical concerns. Resorbable patient-specific β-TCP scaffolds offer advantages over those materials [10]. The material properties can be tailored to meet specific requirements, for example by choice of pore size and microarchitecture, material composition, or coating with additives such as stem cells or growth factors, as demonstrated in preclinical studies and case series [11–13]. As with all alloplastic materials, β-TCP helps to avoid the disadvantages of autografts (donor-site morbidity, unpredictable resorption), allografts, and xenografts (risk of infectious disease transmission, porcine or bovine origin) [14–16]. Covering the scaffold with a collagen membrane further supports the barrier function during CBR and helps to limit graft exposure during wound healing [17]. Histological examination demonstrated the osteoconductive capabilities of the β-TCP scaffold in our case, which is consistent with previous studies [18].

However, there are potential disadvantages to using patient-specific β-TCP scaffolds, such as increased cost and technical requirements for design and fabrication. β-TCP scaffolds are brittle and therefore cannot be fixed with conventional titanium screws drilled directly through the implant. In the present case, the scaffold was secured with poly-lactide pins (SonicWeld® system) because of their ease of application and their documented use in ridge augmentation procedures [19]. These limitations may be overcome by further development of β-TCP material properties. Data is still scarce on the risk of dehiscence with larger β-TCP scaffolds.

Healing time with alloplastic materials may be longer than with autologous bone alone. In the present case, the dental implant was placed approximately ten months after the initial surgery to allow sufficient bone formation before re-entry [20, 21]. This makes the method inferior for patients seeking rapid dental rehabilitation. However, this ultra-slow resorption and remodeling rate can be advantageous in situations where implant placement will be delayed by some time after augmentation. The β-TCP scaffolds’ volume stability over a longer period allows more flexible timing of implant placement compared to allografts, where resorption will happen within a few months after surgery, and implants must be placed in this shorter time window. In the presented case, upon re-entry ten months after augmentation, a substantial portion of the scaffold was still discernible. Further gradual resorption is anticipated to occur over time, and the exact anatomic fit and maintenance of peri-implant health mitigate concerns associated with the presence of residual β-TCP.

This case report provides evidence of the feasibility and safety of using patient-specific β-TCP scaffolds for alveolar ridge augmentation. The use of patient-specific scaffolds is particularly beneficial in challenging cases where traditional ridge augmentation techniques may reach their limit. Given the proof-of-concept nature of this study, a straightforward patient case was selected. The next step towards clinical integration of the presented technique could be CBR for more extensive horizontal and vertical bone regeneration in the esthetic (anterior) region and in combination with soft-tissue augmentation. Future studies on biocompatibility, long-term stability, and the use of β-TCP in combination with other techniques and materials are desirable. New and improved manufacturing methods and the integration of patient-specific β-TCP scaffolds with digital planning technologies will be important areas of future research.

In conclusion, the use of patient-specific β-TCP scaffolds for alveolar ridge augmentation showed successful results in terms of esthetics and function as well as ease of surgical procedure in this case report demonstrating a medium-sized alveolar ridge defect, with high satisfaction of both the patient and the clinician. Patient-specific β-TCP scaffolds offer a promising approach for restoring complex bony defects in the oral cavity, with the potential for improved predictability and reduced morbidity compared to other materials and augmentation techniques. Based on these results, it can be recommended to consider this novel use of patient-specific β-TCP scaffolds for customized bone regeneration (CBR) in appropriate cases as an alternative option for alveolar ridge augmentation, preferably in the context of further clinical studies with larger sample sizes.

### Electronic supplementary material

Below is the link to the electronic supplementary material.

Supplementary Material 1

## Data Availability

Data sharing is not applicable to this article as no datasets were generated or analyzed during the current study

## Abbreviations

β-: TCP beta-tricalcium phosphate
CAD/CAM computer: aided design and manufacturing
CBCT cone: beam computed tomography
CBR: customized bone regeneration
GBR: guided bone regeneration
