# Supplementary material for: Patient-specific beta-tricalcium phosphate scaffold for customized alveolar ridge augmentation: a case report: Case Report: patient-specific β-TCP scaffold for alveolar ridge CBR
Source: Int J Implant Dent. 2024 May 1;10:21. doi: 10.1186/s40729-024-00541-2 (PMC11063008; doi:10.1186/s40729-024-00541-2)
Supplement: Supplementary file 1 — Supplementary Material 1 [file 40729_2024_541_MOESM1_ESM.pdf]

Patient-specific Beta-Tricalcium Phosphate Scaffold for Customized Alveolar Ridge Augmentation: A Case Report

Schönegg D., Essig H., Al-Haj Husain A., Weber F.E., Valdec S.

Introduction

novel patient-specific CAD/CAM scaffold for bone regeneration with beta-tricalcium phosphate (β-TCP)

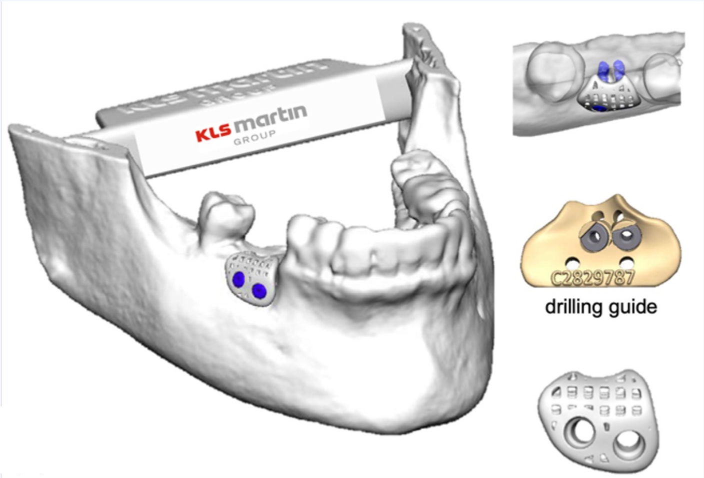

designed to meet anatomic and functional requirements

Case Report

extensive horizontal bone loss: two-stage implant therapy concept

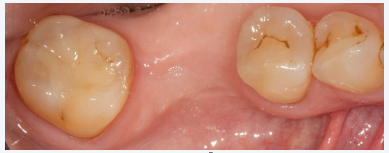

augmentation

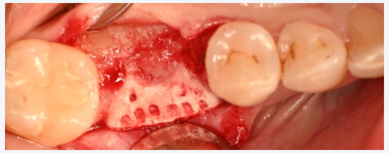

implant placement

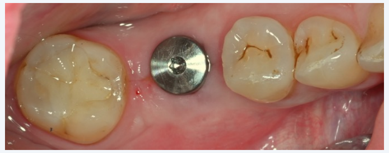

successful single-crown reconstruction

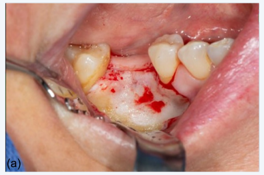

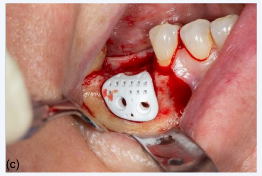

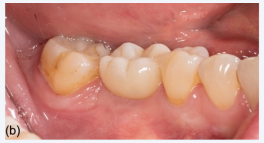

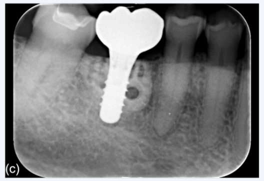

Results

advantages of β-TCP scaffolds:

- precise customized planning
- avoidance of xenogeneic (porcine) materials
- avoidance of allogeneic materials
- avoidance of donor site morbidity
- material properties tailored to meet specific requirements

osteoconductive capabilities: newly formed cancellous bone

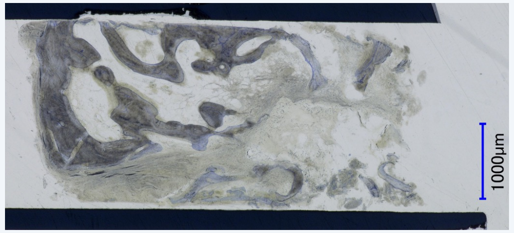

Conclusion

CAD/CAM β-TCP scaffolds are a promising alternative for precise, predictable and effective alveolar ridge augmentation.
